# Need for recovery and different types of early labour force exit: a prospective cohort study among older workers

**DOI:** 10.1007/s00420-019-01404-9

**Published:** 2019-02-11

**Authors:** D. Stynen, N. W. H. Jansen, J. J. M. Slangen, A. de Grip, IJ. Kant

**Affiliations:** 1 Department of Epidemiology, CAPHRI School for Public Health and Primary Care, Faculty of Health, Medicine and Life Sciences, Maastricht University, P.O. Box 616, 6200 MD Maastricht, The Netherlands; 2 Research Centre for Education and the Labour Market (ROA), Maastricht University, Maastricht, The Netherlands

**Keywords:** Older workers, Need for recovery, Early labour force exit, Retirement intentions

## Abstract

**Purpose:**

This study examines the relationship between need for recovery (NFR) and labour force exit (LFE) among older workers. Different types of LFE (early retirement, work disability and unemployment) are considered, and the role of potential confounding and modifying factors, including the availability of early LFE schemes, is examined. Also, associations between NFR and the intention and ability to prolong one’s working life, which are known determinants of LFE, are assessed.

**Methods:**

A subsample of older workers from the Maastricht Cohort Study was examined (*n* = 2312). The relationship between NFR and LFE was investigated by means of Cox regression analyses. Logistic regression analyses were performed to investigate cross-sectional associations between NFR and the intention and ability to prolong working life.

**Results:**

Elevated NFR was associated with a higher risk of overall LFE during a 4-year follow-up period (HR 1.39, 95% CI 1.09–1.78), and specifically with a higher risk of leaving the labour force through early retirement and work disability. When early retirement schemes were available, strong and significant associations between NFR and LFE were observed (HR 2.79, 95% CI 1.29–6.02), whereas no significant associations were found when such schemes were unavailable. Older workers with a higher NFR also had earlier retirement intentions and lower self-assessed abilities (both physical and mental) to prolong their working life until the mandatory retirement age.

**Conclusions:**

Because this study shows that NFR is a precursor of LFE among older workers, monitoring NFR is important for timely interventions aimed at reducing NFR to facilitate extended labour participation.

**Electronic supplementary material:**

The online version of this article (10.1007/s00420-019-01404-9) contains supplementary material, which is available to authorized users.

## Introduction

Due to demographic shifts, the work force in many industrialized countries is characterized by a growing proportion of older workers approaching eligible retirement age (Organisation for Economic Co-operation and Development 2014). Increased longevity further undermines the sustainability of pension systems worldwide (van Soest and Vonkova 2014). Consequently, public policies are aimed at increasing the labour participation of older workers and extending the eligible retirement age (de Grip et al. 2013). Such measures have resulted in a gradual increase in the average Dutch retirement age from 61.0 years in 2007 to 64.8 years in 2017 (Statistics Netherlands 2018a). However, compared to younger workers, the labour force participation of older workers—defined as workers aged 45 years or older (World Health Organization 1993)—still remains at a lower, sub-optimal level (Statistics Netherlands 2018b). Retirement can be regarded as a transition between work roles (like turnover or changing occupations), involving a process that workers undergo and in which an individual’s ability, motivation and opportunity to work play a role (Forrier et al. 2009; de Wind et al. 2015). Despite similarities across career transitions, the specific predictors of transitions like early retirement and turnover are, to an important extent, different (Adams and Beehr 1998). Earlier studies on the specific factors affecting decisions like early retirement in older workers have stressed its multi-factorial etiology, involving factors from different domains (de Wind et al. 2014). A first important factor stems from the personal domain, as Robroek et al. (2013) and de Wind et al. (2014) found that poor health was associated with a higher risk of earlier exit from paid employment. Second, several work-related factors have been associated with decreased labour participation, including high psychological job demands (Canivet et al. 2013), low job control (Robroek et al. 2013) and low appreciation at work (de Wind et al. 2014). In addition, demographic factors, such as a low education level, have been associated with a higher risk of early exit from paid employment (Robroek et al. 2015). Although our understanding of the determinants of labour force exit (LFE) is growing, it will in practice be challenging to identify older workers with an elevated risk for LFE, as many older workers are simultaneously exposed to multiple hindering or facilitating factors, and insight into possible indicators of where an older worker is in the process of LFE is still sparse (de Wind et al. 2015).

The work load–work capacity model of van Dijk et al. (1990) may be useful in identifying ‘precursors’ of emergent LFE. According to this model, long-term impacts on older workers’ health and labour participation result from an imbalance between the work load (e.g. exposure to high psychological or physical job demands) and workers’ carrying capacity (e.g. low job control, poor health, work motivation, and lack of skills) to manage these demands (Heerkens et al. 2004). The model also suggests that long-term effects occur via a pathway of short-term effects involving insufficient recovery from work (van Veldhoven and Broersen 2003; Josten and Schalk 2010).

The concept of NFR represents the short-term effects of a work day and has been defined as the need to recuperate from work-induced fatigue, generally experienced after a day of work (Jansen et al. 2002). The concept involves the intensity of work-induced fatigue, both mental and physical, as well as the time required to return to a normal or pre-stressor level of functioning. Employees with elevated NFR are characterized by temporary feelings of overload, lack of energy for new effort and reduced performance (van Veldhoven 2008). Elevated NFR might hinder labour participation among older workers, as it was identified as a risk factor for work-related outcomes such as absence due to sickness (de Croon et al. 2003), occupational disability (Otten et al. 2012), reduced working hours (de Raeve et al. 2009) and early retirement intentions (Oude Hengel et al. 2012). Also, NFR was considered a possible link in the pathway between high physical job demands and the risk of losing employment in older workers (Gommans et al. 2016). However, to our knowledge, direct effects of NFR on LFE of older workers have not been investigated. The present study addresses this omission and primarily aims to identify NFR as a potential precursor of LFE among older workers, making NFR an important indicator in occupational health surveillance concerned with the labour market participation of older workers. In our study, different types of LFE, including early retirement, work disability or becoming unemployed will be distinguished because, aside from pure early retirement routes, some older workers settle into retirement through a (longer) period of work inability or unemployment (Schils 2008). Omitting these alternatives—which may act as complements or substitutes—from analysis may therefore result in an underestimation of the group of older workers at risk for permanent LFE. To enhance our understanding of this subject, three additional considerations will be taken into account. First, as indicated earlier, both NFR and LFE might be influenced independently by different demographic, work environment and personal factors, and may therefore also influence the developing relationship between NFR and LFE over time. As the latter association has not been investigated earlier, we take an explorative stance to investigate this issue by including relevant confounding and modifying factors when assessing the association between NFR and LFE, or by performing stratified analyses, respectively.

Second, beside demographic, work environment and personal factors, the association between NFR and LFE might be influenced by contextual factors on a national (e.g. social security system) and organizational level (e.g. availability of early retirement schemes), since they might affect employees’ LFE decisions. Whereas the determinants of LFE discussed earlier mainly have to do with workers’ ability and motivation to work, the factors that are discussed here, rather, involve the opportunity structure surrounding LFE. Different associations might be observed between those older workers employed in companies in which early retirement schemes are available and those employed in companies in which such schemes are unavailable. Therefore, this study will also examine the role of such schemes in an explorative way.

Finally, in this study, we also investigate associations between NFR and intentions and perceived ability to prolong working life. On the one hand, the intentions and perceived ability of employees to prolong their working life might be less prone to contextual opportunities for LFE as compared to actual LFE (van Soest et al. 2007). On the other hand, as we have positioned NFR in the pathway to LFE, investigating its associations with other key determinants involving individuals’ appraisals of their ability and motivation to work may add further insight into older workers’ retirement decision-making processes (de Wind et al. 2015).

In sum, the aim of this study is to prospectively investigate the relationship of NFR with overall and specific types of LFE, as well as its associations with workers’ intentions and ability to prolong working life among a sample of older Dutch workers, while taking into account factors from the demographic, work environment and personal domains as well as the availability of opportunities for LFE within companies.

## Methods

### Study population

Data from the Maastricht Cohort Study were used. This is an ongoing prospective cohort established in May 1998, which included 12,140 respondents from 45 different companies at baseline measurement. Data were collected by means of self-administrated questionnaires. The design of the cohort is described elsewhere in detail (Kant et al. 2003; Mohren et al. 2007). The study was conducted in accordance with the ethical standards laid down in the 1964 Declaration of Helsinki and its later amendments.

In this study, follow-up wave October 2008 (*n* = 6082) was considered the starting point for the analyses, and two follow-up waves (October 2012 and October 2014) were included, resulting in a total follow-up duration of 6 years. At wave 2012, *n* = 5894 questionnaires were sent out, and *n* = 4783 questionnaires were returned, resulting in a response rate of 81%. At wave 2014, *n* = 3450 questionnaires were sent out to those who were not fully retired at wave 2012. A total of *n* = 2945 questionnaires was returned (response rate of 85%). All respondents who indicated they were fully retired at wave 2012 received a specific questionnaire assessing only questions on retirement at wave 2014 (*n* = 1284 sent out, response rate 90%) and were therefore not eligible for inclusion. Included in the study population were all respondents aged 45–59 years who indicated they were employed at wave 2008 (*n* = 3932). Employees aged 59 years and older were excluded (*n* = 349), as they could reach the mandatory retirement age of 65 years during the follow-up period. Workers under the age of 45 years were not considered older workers and were therefore excluded (*n* = 981). Those who were employed but also indicated they were (partially) receiving disability insurance or unemployment benefits, were retired and/or were not involved in paid employment, were excluded at wave 2008 (*n* = 32). Also excluded at wave 2008 were those with multiple jobs (*n* = 118), as well as those who were employed but (partly) disabled for work, on sabbatical leave, on pregnancy or parental leave (*n* = 120) or were self-employed (*n* = 20). This resulted in a total study population of 2312 employees at wave 2008.

### Need for recovery

Need for recovery was measured at wave 2008 and was assessed using a subscale from the Dutch Questionnaire on the Experience and Evaluation of Work (QEEW) (van Veldhoven and Broersen 2003). This scale is composed of 11 dichotomous items (yes/no), which represent the short-term effects of a working day, and scores range from 0 to 11. Example items are: ‘At the end of a working day I am really feeling worn out’, ‘I find it hard to relax at the end of a working day’ and ‘When I get home, people should leave me alone for some time’. The subscale score was transformed into a scale ranging from 0 to 100: a higher score indicates a higher NFR. This transformation is in line with the QEEW procedure to increase comparability across subscales initially measured on different scale points. Thus, irrespective of the subscale, ‘0’ indicates the most desirable score and ‘100’ indicates the most undesirable score. The reliability of the scale is good (Cronbach’s alpha of 0.90). A cutoff point of 6 (out of 11 items) was used to define employees with an elevated NFR, as previous research showed that workers who score at this point have a higher risk of developing psychological complaints than those who score below that cutoff point (Broersen et al. 2004; van der Starre et al. 2013). Employees scoring below the cutoff point were considered to have a low–medium NFR, employees with a score of 6 or higher were considered to have a high NFR (the latter also referred to as ‘NFR caseness’).

### Labour force exit

Labour force exit was assessed at waves 2012 and 2014. Respondents were asked to provide an overview of their work status(es) since the preceding questionnaire. This means that at wave 2012, employees described their work status(es) from October 2008 through October 2012; and at wave 2014, employees described their work status(es) from October 2012 through October 2014. First, the current work status of the employee was assessed. Respondents could indicate that they were currently employed, self-employed, receiving disability insurance, receiving unemployment benefits, retired and/or not involved in paid employment. At wave 2014, respondents could additionally indicate if they were actively seeking employment. For each work status, employees indicated whether this was fully or partly applicable. If changes in work status had taken place since the preceding questionnaire, employees were asked to describe their earlier work status(es) using the items listed above. For each specific work status, the start and end time were requested by month and year.

Employees were classified as having exited the labour force early if they indicated non-employment status (i.e. disability insurance, unemployment benefits, retirement, no paid employment and/or actively seeking employment) at any point during the follow-up period and were not involved in any type of (self) employment.

Beside full LFE, it is possible to leave the labour force gradually, for instance by phased retirement combined with part-time employment. Therefore, in the analyses investigating the specific types of LFE, employees were also considered to have exited the labour force early if they indicated reasons for current non-employment and reasons for being partly (self)employed simultaneously. Employees were classified as ‘retired early’ if they indicated they were fully or partly retired during any follow-up period. Employees who indicated receiving any type of disability insurance during follow-up were classified as being disabled for work, and employees who indicated receiving any type of unemployment benefits during follow-up were classified as being unemployed.

### Retirement intentions and ability to prolong working life

Items on intentions to prolong working life were first assessed at wave 2012 and were based on a study by van Dam et al. (2009). The first item measured an employee’s intention to prolong working life until reaching the mandatory retirement age: ‘It is my intention to keep working until I reach the mandatory retirement age’. A second item, measuring an employee’s intention to prolong working life beyond the mandatory retirement age, was assessed with a similar statement. The ability of employees to prolong working life was assessed with two items based on a Koppes et al. (2011) study. The first item assessed the physical ability to prolong working life: ‘I think I am physically able to work in my current job until reaching the mandatory retirement age’. The second item assessed mental ability with a similar statement. All four items were scored on a five-point Likert scale, with response options ranging from ‘strongly agree’ to ‘strongly disagree’. These five response categories were recoded into two categories; a ‘(strongly) agree’ category and a ‘neutral–(strongly) disagree’ category.

### Early labour force exit schemes

Access to LFE schemes was first assessed at wave 2012. Employees were asked to indicate ‘whether within the company at which you are currently employed, schemes exist which facilitate labour force exit before reaching the mandatory retirement age’. Response options were ‘no’, ‘yes’, ‘not applicable’ and ‘don’t know’. Employees indicating that such schemes exist within their company were asked to indicate at which age they were eligible to make use of these schemes.

### Confounding and modifying factors

All factors were measured at wave 2008, except for education level. When analyses concern another baseline (i.e. 2012), all confounders for these analyses were assessed at that wave. If possible, for descriptive and stratification purposes, factors were further recoded into categorical variables. Education level was measured at wave 1998 and recoded into three levels: low (primary school, lower vocational education), medium (lower secondary school, intermediate vocational school, upper secondary school) or high (higher vocational school, university). Our measure for education level therefore reflects educational attainment at labour market entry. The number of working hours per week was measured by one item that included response options > 40, 36–40, 26–35, 16–25 or < 16 h per week, which were recoded into three categories: ≥ 36, 26–35 or ≤ 25 h per week. To assess work schedule, employees first indicated whether they were engaged in day or shift work. Next, employees performing shift work could indicate the specific type of shift in which they were engaged. Employees who indicated they were engaged in shift work and/or indicated a specific shift type were classified as shift workers and all other employees were classified as day workers. For the purpose of stratification, age was also recoded in three categories: 45–49, 50–54 and 55–59 years. Psychological job demands were measured with a scale from the validated Dutch version of the Job Content Questionnaire (JCQ) (Karasek 1985) (Cronbach’s alpha of 0.71). To measure decision latitude, two subscales from the JCQ were combined: skill discretion and decision authority (Cronbach’s alpha of 0.80). The total scores for the psychological job demands (range 12–48) and decision latitude (range 24–96) subscales were grouped into tertiles of low, medium and high levels of psychological job demands and decision latitude for the stratified analyses. Supervisor social support was measured with a subscale of the JCQ, resulting in a range of 4–16 (Cronbach’s alpha of 0.86). The total score of this subscale was dichotomized at the median, resulting in low and high supervisor social support for the stratified analyses. Self-perceived health was measured with one item from the SF-36 Health Survey (Aaronson et al. 1998): ‘How would you rate your health in general?’. In line with an earlier study by Dalstra et al. (2002), the response options ‘excellent’, ‘very good’ and ‘good’ were grouped into ‘good health’, and ‘moderate’ or ‘bad’ were grouped into ‘moderate–poor health’. To assess spousal retirement, employees first indicated whether they currently have a partner (yes/no). Employees who indicated that they currently have a partner were asked to indicate whether their partner was currently retired (yes/no). These items were recoded into two categories: a ‘retired spouse’ category, and a ‘non-retired spouse’ category that included employees with a currently employed partner and employees without a partner. Attachment to work for financial resources was assessed with a self-formulated item: ‘There are several reasons for employment. Could you indicate to which extent financial resources determine your level of attachment to work?’ Response options included: ‘very attached’, ‘attached’, ‘somewhat attached’ and ‘not attached’ to work. The two latter response options were recoded into one category: ‘not to somewhat attached’.

### Statistical analysis

First, the prevalence of the baseline demographic, work environment and personal characteristics and the prevalence of NFR caseness in the study population at study baseline were described. The distribution of NFR was skewed to the right, so Poisson regression analyses were conducted and pairwise comparisons were investigated by means of the Wald Chi square test to test for differences in the mean NFR scores for all characteristics separately.

The relationship between NFR and overall LFE was investigated by means of Cox regression analyses. In all analyses, both NFR caseness and the continuous NFR score were investigated. Four models were investigated: a crude model (model 1), a model adjusting for demographic confounders (model 2), a model additionally adjusting for confounders of the work environment (model 3) and a model additionally adjusting for confounders from the personal domain (model 4). To investigate the role of these factors as effect-modifiers, results were stratified for each characteristic from these three domains. Hazard ratios (HR) and 95% confidence intervals (CI) were reported. The potential moderating role of all characteristics was tested stepwise by adding the interaction term—based on the raw scores—between a characteristic and NFR scores or NFR caseness to the Cox regression analysis, respectively, whenever the HR was significant in at least one of the strata under consideration, for all models.The role of access to early retirement schemes in the risk of overall LFE was investigated with Cox regression analysis. The starting point for this analysis was the 2012 wave, which is when such schemes were first assessed. Here, the same inclusion and exclusion criteria as mentioned earlier were used, except that the information of the 2012 wave was used and criteria applied to the 2012 respondents and only employees aged 60–63 years were included. The investigation of this age category was justified because the majority of employees in the Netherlands (within our study population, this majority was 89%) have to be 60 years or older to be eligible for LFE schemes (Euwals et al. 2011). Employees who indicated ‘not applicable’ or ‘don’t know’ on the item regarding the availability of LFE schemes were excluded. This resulted in a study population of 204 employees. The four models mentioned above were investigated, and all confounders for this analysis were assessed at wave 2012. Next, associations between NFR and specific types of LFE (risk of early retirement, becoming disabled for work and unemployment) were separately investigated by means of Cox regression. For all three specific types of LFE, the four models were investigated.

For all Cox regression analyses performed, time-to-event was modelled monthly, based on the month and year an employee indicated that an event (e.g. losing employment) had occurred for the first time. Employees who reported that an event had occurred before or after the investigated follow-up period (e.g. a forthcoming early retirement date) were excluded for that particular analysis. The proportional hazard assumption was investigated for all Cox regression models and was met in the large majority of the models. As the intention and ability to prolong working life were first assessed at wave 2012, this wave was considered the starting point for these analyses. The same inclusion and exclusion criteria as mentioned earlier were used, except that these criteria were based on the 2012 respondents, inevitably resulting in a somewhat smaller study population (*n* = 1607). Cross-sectional associations between NFR and intention and ability to prolong working life were investigated by means of logistic regression analyses. Odds ratios (OR) and 95% CI were reported. For all items, the odds of agreeing with the statement were investigated: a larger OR implies higher odds of agreeing with the statement. To analyse the data, IBM SPSS Statistics 23.0 was used, and a *p* value of < 0.05 was considered statistically significant. Significant coefficients (HR and OR) have been marked in bold in all tables. If the lower limit of a 95% CI in bold contains 1.00, the lower limit is ≥ 1.001 and the corresponding *p* value is < 0.05. If the upper limit of a 95% CI in bold contains 1.00, the upper limit is ≤ 0.999 and the corresponding *p* value is < 0.05.

### Results

### Descriptives

The baseline characteristics of the study population are presented in Table 1. The table demonstrates statistically significant differences in mean NFR scores among different demographic, work environment and personal factors.

**Table 1:** Description of and mean NFR scores according to demographics, work and personal characteristics of the study population at baseline October 2008 (*n* = 2312)

	(%)		NFR mean score (0–100)	*p* value
Demographics				
Gender				
Male	72.7		32.57	0.860
Female	27.3		32.29	
Age				
45–49 years	36.2		29.51	0.016 (45–49 vs. 50–54)
50–54 years	36.8		33.51	0.387 (50–54 vs. 55–59)
55–59 years	27.1		35.14	0.002 (45–49 vs. 55–59)
Education level				
Low	15.2		36.17	0.014 (low vs. medium)
Medium	43.9		30.67	0.121 (medium vs. high)
High	40.8		33.10	0.177 (high vs. low)
Work environment				
Number of working hours per week				
≥ 36	71.0		32.78	0.457 (≥ 36 vs. 26–35)
26–35	17.6		34.26	0.005 (26–35 vs. ≤25)
≤ 25	11.5		26.78	0.005 (≥ 36 vs. ≤25)
Work schedule				
Shift work	17.9		36.70	0.009
Day work	82.1		31.48	
Psychological job demands				
Low	43.2		22.13	< 0.0001 (low vs. medium)
Medium	30.8		33.92	< 0.0001 (medium vs. high)
High	26.0		48.14	< 0.0001 (high vs. low)
Decision latitude				
Low	29.6		37.57	0.002 (low vs. medium)
Medium	36.2		31.96	0.048 (medium vs. high)
High	34.1		28.67	< 0.0001 (high vs. low)
Supervisor support				
Low	41.5		38.74	< 0.0001
High	58.5		28.18	
Personal and health				
Self-perceived health				
Moderate-poor	12.9		57.94	< 0.0001
Good	87.1		28.72	
Spousal retirement				
Yes	2.2		32.17	0.433
No	97.8		36.26	
Attachment to work for financial resources				
Very attached	37.3		36.79	< 0.0001 (very attached vs. attached)
Attached	56.0		30.45	0.058 (attached vs. not-somewhat attached)
Not–somewhat attached	6.6		25.46	< 0.0001 (very attached vs. not-somewhat attached)
Need for recovery caseness				
No	74.6		N/A	
Yes	25.4			

*N/A* not applicable

### Risk of early labour force exit

In Table 2, the results on the association of NFR with overall risk of LFE are presented. In models 1 and 2, NFR caseness was found to be associated with a higher risk of LFE (in model 2: HR 1.29, 95% CI 1.01–1.65; *p* < 0.05). The NFR continuous scores remained statistically significantly associated with a higher risk of LFE throughout all models (in model 4: HR 1.01, 95% CI 1.00–1.01; *p* < 0.05). The effect modification on these associations by significant demographic, personal and work environment factors was tested, but no significant interactions were found. However, when stratifying for different demographic characteristics, different associations between NFR and LFE were observed. The NFR was a statistically significant risk factor for LFE among men, but not among women. Different associations between NFR and LFE were also observed among the different work environment characteristics. Among employees reporting low levels of supervisor social support, NFR was statistically significantly associated with LFE, whereas such findings were not observed among employees with high levels of supervisor social support. In the personal domain, NFR was a statistically significant risk factor for LFE among employees who did not have a retired spouse, whereas such associations were not observed among employees who did have a retired spouse.

**Table 2:** Hazard ratios and 95% confidence intervals for the chance of early labour force exit according to NFR caseness and NFR continuous score (October 2008–October 2014)

	Model 1			Model 2		Model 3		Model 4	
	HR		95% CI	HR	95% CI	HR	95% CI	HR	95% CI
Total study population (*n* = 2292)									
NFR caseness	**1.39**		**1.09–1.78**	**1.29**	**1.01–1.65**	1.24	0.94–1.62	1.22	0.92–1.62
NFR continuous score	**1.01**		**1.00–1.01**	**1.01**	**1.00–1.01**	**1.00**	**1.00–1.01**	**1.01**	**1.00–1.01**
*Demographics*									
Gender^a^									
Men (*n* = 1651)									
NFR caseness	**1.46**		**1.11–1.93**	**1.38**	**1.04–1.81**	1.33	0.98–1.79	1.30	0.94–1.78
NFR continuous score	**1.01**		**1.00–1.01**	**1.01**	**1.00–1.01**	**1.01**	**1.00–1.01**	**1.01**	**1.00–1.01**
Women (*n* = 623)									
NFR caseness	1.10		0.64–1.91	0.96	0.55–1.67	0.81	0.43–1.51	0.78	0.40–1.56
NFR continuous score	1.00		1.00–1.01	1.00	0.99–1.01	1.00	0.99–1.01	1.00	0.99–1.01
Age category^a^									
45–49 years (*n* = 831)									
NFR caseness	**1.79**		**1.01–3.17**	1.78	1.00–3.16	1.73	0.93–3.20	1.67	0.88–3.17
NFR continuous score	1.00		1.00–1.02	1.01	1.00–1.02	1.01	1.00–1.02	1.01	1.00–1.02
50–54 years (*n* = 843)									
NFR caseness	1.12		0.66–1.88	1.15	0.68–1.91	1.05	0.58–1.88	1.10	0.60–2.02
NFR continuous score	1.00		0.99–1.01	1.00	1.00–1.01	1.00	0.99–1.01	1.00	0.99–1.01
55–59 years (*n* = 618)									
NFR caseness	1.21		0.88–1.67	1.20	0.87–1.66	1.16	0.82–1.65	1.13	0.78–1.65
NFR continuous score	**1.00**		**1.00–1.01**	1.00	1.00–1.01	1.00	1.00–1.01	1.00	1.00–1.01
Education level^a^									
Low (*n* = 336)									
NFR caseness	1.38		0.76–2.51	1.47	0.81–2.67	1.89	0.90–3.98	1.95	0.88–4.33
NFR continuous score	1.00		0.99–1.01	1.00	1.00–1.01	1.01	1.00–1.02	1.01	1.00–1.02
Medium (*n* = 987)									
NFR caseness	**1.51**		**1.04–2.20**	1.35	0.93–1.97	1.26	0.84–1.90	1.29	0.83–1.99
NFR continuous score	**1.01**		**1.00–1.01**	**1.01**	**1.00–1.01**	1.01	1.00–1.01	1.01	1.00–1.01
High (*n* = 921)									
NFR caseness	1.31		0.89–1.93	1.11	0.75–1.65	1.06	0.70–1.61	0.99	0.63–1.55
NFR continuous score	1.01		1.00–1.01	1.00	1.00–1.01	1.00	1.00–1.01	1.00	1.00–1.01
*Work environment*									
Number of working hours per week^a^									
≥36 (*n* = 1584)									
NFR caseness	**1.45**		**1.09–1.92**	**1.34**	**1.00–1.78**	1.30	0.96–1.78	1.23	0.88–1.72
NFR continuous score	**1.01**		**1.00–1.01**	**1.01**	**1.00–1.01**	**1.01**	**1.00–1.01**	1.00	1.00–1.01
26–35 (*n* = 390)									
NFR caseness	1.58		0.93–2.69	1.34	0.77–2.32	1.31	0.73–2.36	1.48	0.80–2.74
NFR continuous score	1.01		1.00–1.01	1.00	1.00–1.01	1.00	0.99–1.01	1.00	1.00–1.01
≤ 25 (*n* = 256)									
NFR caseness	0.23		0.03–1.69	0.29	0.04–2.22	0.21	0.02–1.85	0.24	0.03–2.06
NFR continuous score	1.00		0.99–1.02	1.01	0.99–1.02	1.01	0.99–1.03	1.01	0.99–1.03
Work schedule^a^									
Shift work (*n* = 396)									
NFR caseness	1.26		0.70–2.26	1.17	0.65–2.11	1.13	0.58–2.20	1.06	0.51–2.19
NFR continuous score	1.00		1.00–1.01	1.00	1.00–1.01	1.00	1.00–1.01	1.00	0.99–1.01
Day work (*n* = 1839)									
NFR caseness	**1.47**		**1.12–1.92**	**1.34**	**1.02–1.76**	1.30	0.96–1.74	1.28	0.94–1.76
NFR continuous score	**1.01**		**1.00–1.01**	**1.01**	**1.00–1.01**	**1.01**	**1.00–1.01**	**1.01**	**1.00–1.01**
Psychological job demands^a^									
Low (*n* = 973)									
NFR caseness	1.42		0.93–2.18	1.30	0.84–2.01	1.22	0.78–1.90	1.21	0.76–1.94
NFR continuous score	1.01		1.00–1.01	1.01	1.00–1.01	1.00	1.00–1.01	1.00	1.00–1.01
Medium (*n* = 690)									
NFR caseness	1.50		0.93–2.40	1.38	0.85–2.22	1.34	0.82–2.19	1.41	0.83–2.38
NFR continuous score	**1.01**		**1.00–1.02**	**1.01**	**1.00–1.02**	**1.01**	**1.00–1.01**	**1.01**	**1.00–1.02**
High (*n* = 584)									
NFR caseness	**1.62**		**1.03–2.54**	1.34	0.85–2.13	1.19	0.73–1.94	1.13	0.67–1.92
NFR continuous score	**1.01**		**1.00–1.01**	1.01	1.00–1.01	1.00	1.00–1.01	1.00	0.99–1.01
Decision latitude^a^									
Low (*n* = 668)									
NFR caseness	1.28		0.86–1.91	1.20	0.80–1.80	1.24	0.79–1.94	1.21	0.75–1.96
NFR continuous score	1.00		1.00–1.01	1.00	1.00–1.01	1.00	1.00–1.01	1.00	1.00–1.01
Medium (*n* = 819)									
NFR caseness	1.46		0.99–2.16	1.24	0.84–1.84	1.28	0.84–1.95	1.20	0.76–1.88
NFR continuous score	**1.01**		**1.00–1.01**	1.01	1.00–1.01	1.01	1.00–1.01	1.01	1.00–1.01
High (*n* = 773)									
NFR caseness	1.29		0.76–2.19	1.24	0.73–2.11	1.27	0.72–2.23	1.32	0.73–2.39
NFR continuous score	1.01		1.00–1.01	1.01	1.00–1.01	**1.01**	**1.00–1.02**	**1.01**	**1.00–1.02**
Supervisor social support^a^									
Low (*n* = 944)									
NFR caseness	**1.54**		**1.07–2.19**	1.38	0.96–1.97	1.32	0.90–1.96	1.34	0.88–2.02
NFR continuous score	**1.01**		**1.00–1.01**	**1.01**	**1.00–1.01**	**1.01**	**1.00–1.01**	**1.01**	**1.00–1.01**
High (*n* = 1330)									
NFR caseness	1.28		0.91–1.81	1.24	0.88–1.75	1.16	0.79–1.68	1.13	0.75–1.69
NFR continuous score	1.00		1.00–1.01	1.00	1.00–1.01	1.00	1.00–1.01	1.00	1.00–1.01
*Personal characteristics*									
Self-perceived health^a^									
Moderate–poor (*n* = 293)									
NFR caseness	1.06		0.59–1.91	1.03	0.56–1.87	1.03	0.53–2.00	0.80	0.40–1.63
NFR continuous score	1.00		0.99–1.01	1.00	0.99–1.01	1.00	0.99–1.01	1.00	0.99–1.01
Good (*n* = 1995)									
NFR caseness	**1.43**		**1.09–1.89**	**1.33**	**1.01–1.76**	1.27	0.93–1.72	1.31	0.96–1.79
NFR continuous score	**1.01**		**1.00–1.01**	**1.01**	**1.00–1.01**	**1.01**	**1.00–1.01**	**1.01**	**1.00–1.01**
Attachment to work for financial resources^a^									
Not-somewhat attached (*n* = 153)									
NFR caseness	0.91		0.35–2.38	1.00	0.37–2.69	1.28	0.45–3.67	1.28	0.36–4.53
NFR continuous score	1.00		0.99–1.01	1.00	0.99–1.01	1.00	0.99–1.02	1.00	0.99–1.02
Attached (*n* = 1281)									
NFR caseness	**1.60**		**1.14–2.24**	**1.47**	**1.05–2.06**	1.31	0.90–1.91	1.34	0.90–1.98
NFR continuous score	**1.01**		**1.00–1.01**	**1.01**	**1.00–1.01**	1.01	1.00–1.01	**1.01**	**1.00–1.01**
Very attached (*n* = 854)									
NFR caseness	1.27		0.87–1.88	1.16	0.79–1.72	1.19	0.78–1.82	1.07	0.68–1.68
NFR continuous score	1.00		1.00–1.01	1.00	1.00–1.01	1.00	1.00–1.01	1.00	1.00–1.01
Spousal retirement^a^									
Yes (*n* = 50)									
NFR caseness	0.75		0.16–3.54	0.45	0.08–2.50	0.08	0.00–1.56	0.36	0.01–13.84
NFR continuous score	1.00		0.98–1.02	1.00	0.98–1.02	0.98	0.95–1.02	1.03	0.98–1.09
No (*n* = 2199)									
NFR caseness	**1.39**		**1.08–1.79**	1.27	0.98–1.64	1.21	0.92–1.61	1.23	0.92–1.64
NFR continuous score	**1.01**		**1.00–1.01**	**1.01**	**1.00–1.01**	**1.00**	**1.00–1.01**	**1.01**	**1.00–1.01**

Statistically significant HRs + 95% CIs are marked in bold. In case the lower limit of a bold marked 95% CI contains 1.00, the lower limit is ≥ 1.001 and the corresponding *p* value is < 0.05

Model 1: crude

Model 2: adjusted for gender, age and education level

Model 3: additionally adjusted for number of working hours per week, work schedule, psychological job demands, decision latitude and supervisor social support

Model 4: additionally adjusted for self-perceived health, spousal retirement and attachment to work for financial resources

^a^The variable of stratification was not adjusted for in the models

Results regarding the role of availability of LFE schemes in the association between NFR and overall LFE are presented in Table 3. No significant effect modification was found for the availability of LFE schemes in the relationship between NFR and overall LFE. However, stratified analyses showed that among employees who indicated that LFE schemes were available, both NFR caseness and a higher NFR continuous score were associated with a statistically significantly higher risk for LFE in models 1 and 2 (for NFR caseness: HR 3.11, 95% CI 1.42–6.80; *p* < 0.05; for NFR continuous score: HR 1.02, 95% CI 1.00–1.03; *p* < 0.05), whereas among employees who indicated that LFE schemes were not available, no statistically significant associations between NFR and LFE were observed in any of the models.

**Table 3:** Hazard ratios and 95% confidence intervals for the chance of LFE according to the availability of schemes for early labour force exit: among employees aged 60–63, according to NFR caseness and NFR continuous score (October 2012–October 2014)

Schemes for LFE available	Model 1				Model 2				Model 3				Model 4			
	No (*n* = 97)		Yes (*n* = 107)		No		Yes		No		Yes		No		Yes	
	HR	95% CI	HR	95% CI	HR	95% CI	HR	95% CI	HR	95% CI	HR	95% CI	HR	95% CI	HR	95% CI
Total study population (*n* = 204)																
NFR caseness	1.31	0.55–3.14	**2.79**	**1.29–6.02**	1.63	0.62–4.26	**3.11**	**1.42–6.80**	1.31	0.39–4.35	1.73	0.70–4.28	1.64	0.45–6.03	2.30	0.80–6.58
NFR continuous score	1.00	0.99–1.02	**1.01**	**1.00–1.02**	1.00	0.99–1.02	**1.02**	**1.00–1.03**	1.00	0.98–1.02	1.00	1.00–1.02	1.00	0.98–1.02	1.01	1.00–1.02

Statistically significant HRs + 95% CIs are marked in bold. In case the lower limit of a bold marked 95% CI contains 1.00 the lower limit is ≥ 1.001 and the corresponding *p* value is < 0.05

Model 1: crude

Model 2: adjusted for gender, age and education level

Model 3: additionally adjusted for number of working hours per week, work schedule, psychological job demands, decision latitude and supervisor social support

Model 4: additionally adjusted for self-perceived health, spousal retirement and attachment to work for financial resources

In Table 4, associations between NFR and specific types of LFE are presented. A higher NFR continuous score was statistically significantly associated with a higher chance of early retirement in all models (in model 4: HR 1.01, 95% CI 1.00–1.01; *p* < 0.05), whereas statistically significant associations between NFR caseness and early retirement were not observed. Both NFR caseness and a higher NFR continuous score were statistically significantly associated with a higher chance of work disability in all four models (in model 4: for NFR caseness: HR 2.76, 95% CI 1.08–7.05; *p* < 0.05; for NFR continuous score: HR 1.02, 95% CI 1.00–1.03; *p* < 0.05). However, no statistically significant associations between NFR caseness, or NFR continuous score and unemployment were observed in any of the models.

**Table 4:** Hazard ratios and 95% confidence intervals for specific types of early labour force exit: early retirement, work disability and unemployment, according to NFR caseness and NFR continuous score (October 2008–October 2014)

Specific type of labour force exit	Model 1		Model 2		Model 3		Model 4	
	HR	95% CI	HR	95% CI	HR	95% CI	HR	95% CI
Early retirement								
NFR caseness	1.37	0.94–2.00	1.29	0.88–1.89	1.31	0.86–1.98	1.22	0.78–1.90
NFR continuous score	**1.01**	**1.00–1.01**	**1.01**	**1.00–1.01**	**1.01**	**1.00–1.01**	**1.01**	**1.00–1.01**
Work disability								
NFR caseness	**2.44**	**1.14–5.22**	**2.39**	**1.11–5.11**	**3.04**	**1.26–7.33**	**2.76**	**1.08–7.05**
NFR continuous score	**1.01**	**1.00–1.03**	**1.01**	**1.00–1.03**	**1.02**	**1.00–1.03**	**1.02**	**1.00–1.03**
Unemployment								
NFR caseness	1.16	0.80–1.67	1.11	0.77–1.61	1.01	0.68–1.51	1.01	0.66–1.54
NFR continuous score	1.00	1.00–1.01	1.00	1.00–1.01	1.00	0.99–1.01	1.00	0.99–1.01

Statistically significant HRs + 95% CIs are marked in bold. In case the lower limit of a bold marked 95% CI contains 1.00 the lower limit is ≥ 1.001 and the corresponding *p* value is < 0.05

Model 1: crude

Model 2: adjusted for gender, age and education level

Model 3: additionally adjusted for number of working hours per week, work schedule, psychological job demands, decision latitude and supervisor social support

Model 4: additionally adjusted for self-perceived health, spousal retirement and attachment to work for financial resources

Table 5 presents cross-sectional associations between NFR and the intention and ability to prolong working life. In model 1, NFR caseness was associated with lower odds of agreeing with the statement ‘It is my intention to keep working until I reach the mandatory retirement age’ (OR 0.77, 95% CI 0.60–0.99; *p* < 0.05). For the NFR continuous score, the odds were also statistically significant in models 1, 2 and 4 (OR 1.00. 95% CI 0.99–1.00; *p* < 0.05), but not in model 3. Both NFR caseness and a higher NFR continuous score were statistically significantly associated with lower odds of agreeing with the statement, ‘I think I am physically able to work in my current job until reaching the mandatory retirement age’ in all four models (in model 4: for NFR caseness: OR 0.50. 95% CI 0.37–0.68; *p* < 0.05; for NFR continuous score: OR 0.99, 95% CI 0.98–0.99; *p* < 0.05). In all four models, both NFR caseness and a higher NFR continuous score were statistically significantly associated with a lower odds of agreeing with the statement ‘I think I am mentally able to work in my current job until reaching the mandatory retirement age’ (in model 4: for NFR caseness: OR 0.49, 95% CI 0.37–0.65; *p* < 0.05; for NFR continuous score: OR 0.99, 95% CI 0.98–0.99; *p* < 0.05). No statistically significant associations between NFR and intentions to prolong working life beyond the mandatory retirement age were observed in any of the models.

**Table 5:** Odds ratios and 95% confidence intervals for the different items on retirement intentions and ability to prolong working life according to NFR caseness and NFR continuous score (October 2012) (*n* = 1607)

	Model 1		Model 2		Model 3		Model 4	
	OR^a^	95% CI	OR	95% CI	OR	95% CI	OR	95% CI
It is my intention to keep working until I reach the mandatory retirement age								
NFR caseness	**0.77**	**0.60–0.99**	0.80	0.62–1.02	0.84	0.64–1.10	0.76	0.58–1.01
NFR continuous score	**1.00**	**0.99–1.00**	**1.00**	**0.99–1.00**	1.00	0.99–1.00	**1.00**	**0.99–1.00**
I think I am physically able to work in my current job until reaching the mandatory retirement age								
NFR caseness	**0.36**	**0.28–0.46**	**0.36**	**0.28–0.47**	**0.45**	**0.34–0.61**	**0.50**	**0.37–0.68**
NFR continuous score	**0.98**	**0.98–0.99**	**0.98**	**0.98–0.99**	**0.99**	**0.98–0.99**	**0.99**	**0.98–0.99**
I think I am mentally able to work in my current job until reaching the mandatory retirement age								
NFR caseness	**0.36**	**0.28–0.46**	**0.37**	**0.28–0.47**	**0.45**	**0.34–0.59**	**0.49**	**0.37–0.65**
NFR continuous score	**0.98**	**0.98–0.99**	**0.98**	**0.98–0.99**	**0.99**	**0.98–0.99**	**0.99**	**0.98–0.99**
It is my intention to keep working beyond the mandatory retirement age								
NFR caseness	0.90	0.61–1.33	0.90	0.61–1.34	0.95	0.61–1.47	0.91	0.57–1.43
NFR continuous score	1.00	1.00–1.01	1.00	1.00–1.01	1.00	1.00–1.01	1.00	1.00–1.01

Statistically significant ORs + 95% CIs are marked in bold. In case the upper limit of a bold marked 95% CI contains 1.00 the upper limit is ≤ 0.999 and the corresponding *p* value is < 0.05

Model 1: crude

Model 2: adjusted for gender, age and education level

Model 3: additionally adjusted for number of working hours per week, work schedule, psychological job demands, decision latitude and supervisor social support

Model 4: additionally adjusted for self-perceived health, spousal retirement and attachment to work for financial resources

^a^OR < 1 implies less agreement with the statement

## Discussion

The aim of this study was to investigate the possibility that NFR is a precursor of older workers’ labour participation. Longitudinal relationships of NFR with (types of) LFE and cross-sectional associations between NFR with intentions and ability to prolong working life were studied. Key factors from the demographic, work environment and personal domains were considered when studying these associations. The main finding of this study was that NFR is associated with an increased probability of overall LFE over time in workers aged 45 years and older. Higher levels of NFR (as a continuous measure) remained significantly related with LFE when adjusted for factors from all domains. However, NFR caseness was not significantly related with LFE when adjustments were made for factors from the work environment and the personal domain. In line with the work load–work capacity model of van Dijk et al. (1990), this finding suggests that long-term effects like LFE may occur via a pathway of short-term effects involving insufficient recovery from work (van Veldhoven and Broersen 2003). In this sense, NFR can be seen as a ‘precursor’ of LFE. Personal, demographic and work environment factors were studied to further enhance insight into the factors that may play a role in the relationship between NFR and LFE. Effect modification could not be formally established. However, from a descriptive statistics perspective, stratified analyses rendered an indication of differences according to some of the moderators. First, associations between NFR and overall LFE were only observed among men, but not among women. Second, findings suggest significant associations between NFR and LFE when older workers are exposed to low supervisor support. However, these associations were only significant when they were not adjusted for additional confounding factors. These findings suggest that the strength of the relationship between NFR and LFE may depend more upon static demographic characteristics like gender, as well as on unfavourable working conditions. Third, although the direction of the association between NFR and overall LFE was similar among employees with and without organizational schemes for LFE, only statistically significant associations between NFR and overall LFE were found only among employees who had such opportunities. Therefore, it is likely that the choice for LFE is at least partially embedded in the broader organizational context. Similar findings in Vickerstaff et al.’s (2004) study demonstrated the influence of organizational policies and practices on the decision to retire early. The finding that associations between NFR and LFE varied according to factors from various domains suggests that we underestimated the true association between NFR and overall LFE; for instance, among employees with an elevated NFR but without opportunities for LFE, actual LFE might not have been feasible. It should be noted that the findings might be affected by the uneven distribution across various strata, and that effect modification could not be formally established in terms of statistically significant differences between strata, warranting a cautious interpretation of these findings.

Furthermore, NFR was found to be differently associated with specific types of LFE. That is, NFR was associated with a higher risk of early retirement and work disability, but not with a higher chance of unemployment. The finding that NFR was associated with work disability is consistent with earlier studies, where NFR was associated with occupational diseases (Otten et al. 2012) and sickness absence (de Croon et al. 2003), which were both found to be associated with a higher risk of disability pension (Kivimäki et al. 2004). However, to our knowledge, the finding that NFR can be regarded as an immediate precursor of types of LFE like early retirement and work disability among older workers is novel in the literature on NFR and retirement. The different associations between NFR and specific types of LFE might also be at least partly ascribed to the voluntariness of the type of exit. Robroek et al. (2013) found that determinants such as poor health were differently associated with involuntary (e.g. unemployment) as compared to more voluntary types of LFE (e.g. early retirement). Also, contextual factors (e.g. firm closure) might play a larger role in the risk of becoming unemployed, which might (slightly) conceal the association between NFR and unemployment.

Furthermore, elevated NFR was also associated with reduced intentions to prolong working life, and employees with elevated NFR considered themselves less physically and mentally able to work until the mandatory retirement age. Although not explicitly tested, NFR might be associated with long-term outcomes such as LFE through an indirect pathway of reduced intention and ability to prolong working life, which is in line with the work load–work capacity model. It should be noted, however, that these analyses are cross-sectional, and a longitudinal time-window may yield different findings.

### Strengths and limitations

An important strength of this study is the large cohort, which allowed for an investigation of longitudinal associations between NFR and specific types of LFE that have not been previously investigated. Concerning NFR, we investigated both continuous NFR scores and NFR caseness. A cutoff score to define cases of elevated NFR may be helpful in determining which workers are at risk for LFE and could be useful to direct interventions. The HRs for continuous NFR levels were often low, with narrow confidence intervals. However, it should be noted that they were measured on a 100-point scale, meaning that with an HR of 1.01, any 10-point increase on the NFR scale is associated with a 10% increase in the risk of LFE. It was useful to distinguish the main types of LFE in terms of early (obligatory) retirement, work disability and (in)voluntary unemployment, as the results for these specific types of LFE differed. Whereas LFE was approached as a dichotomous outcome in this study, this is not necessarily always the case, as gradual types and multiple combinations of types of LFE also exist. Employees can leave the labour force gradually via bridge employment or may become partially disabled for work. A suggestion for future research would be to further differentiate between gradual and full types of LFE when investigating the impact of NFR. Also, possible return to the labour market after LFE was not taken into consideration in this study. Another strength of this study is that various potential confounders and effect-modifying factors were considered to explore the role of these factors in the association between NFR and LFE via adjusted and stratified models. However, it remains uncertain whether adjusting these associations is required and/or desirable, especially since NFR is considered a comprehensive concept in which numerous effects from the psychosocial work environment are accumulated. The results of the adjusted models might be somewhat overcorrected, which may have led to an underestimation of the strength of the relationships. Although stratification has the advantage of unambiguous interpretation and clearly indicated the strength of association between NFR and LFE in specific subgroups, statistically significant differences across strata could not be established. Despite the factors we took into account in this study, residual confounding cannot be ruled out, as it is possible that not all relevant confounders have been included, and some confounders might not have been optimally operationalized. For instance, education level was assessed as the highest education degree workers had obtained as of May 1998. Although educational attainment earlier in life (e.g. at labour market entry) remains an important determinant of labour participation and opportunities (like training) in later life (Edgerton et al. 2012; Fouarge et al. 2013), workers may have gained additional education in later life, which may underestimate the importance of education level in the association between NFR and LFE. In addition, the factor ‘attachment to work for financial resources’ was considered, but it does not fully grasp the employees’ or families’ financial situation in relation to LFE opportunities. In addition, we investigated the role of spousal retirement, but the reference group comprised both employees with a currently employed partner as well as a relatively small group of employees without a partner. Also, the influence of partnership itself on LFE was not examined in this study. Bias may also stem from the multi-level nature of variables like the availability of schemes for early LFE, where reporting may be more similar among employees working in the same organization. However, given the relatively small sample size in relation to the number of organizations (second order) in the analyses on the role of early exit schemes (Table 3), we cannot account statistically for this possible bias. Overall, this study relies only on self-reports, which may have artificially inflated the strength of the observed relationships (i.e. common method bias), in particular with respect to the cross-sectional analyses (Podsakoff et al. 2012). For descriptive and stratification purposes and for reasons of parsimony and statistical power, categorical and continuous variables were occasionally recoded into categorical variables with fewer categories, which may have resulted in loss of information. An equal set of confounders was investigated for all types of LFE. It is possible, however, that different confounders play a role in the associations between NFR and different types of LFE.

To investigate possible short- and long-term effects of NFR on multiple outcomes, different time-windows, study populations and data-analysis techniques were required. For each type of outcome, the time-window, study population and technique were carefully considered, although this might have hindered direct comparisons between the study findings. As the present study excluded all employees aged 59 years and older at study baseline, the association between NFR and (specific types of) LFE has not been investigated among employees who were close to reaching the mandatory retirement age. Therefore, the findings of this study might not be fully applicable and/or generalized to the group of oldest employees. As this approach might have been somewhat conservative, a suggestion for future studies would be to investigate the association between NFR and LFE in a sample of employees close to reaching their mandatory retirement age, as being close to reaching this age might result in different associations between NFR and LFE.

As all companies included in the cohort fall under Dutch legislation, the findings of this study should be considered in the Dutch context. Many European countries try to discourage early retirement and minimize work disability as much as possible, whereas no general policies on unemployment exist. Social policies for the specific types of LFE and retirement regulations vary across countries (Siegrist et al. 2007), possibly affecting the employees’ opportunities for LFE. Furthermore, varying definitions of (specific types of) LFE and mandatory retirement age might be observed among countries, which suggests that LFE in this study should be considered in the national context.

## Conclusion and implications

This study demonstrated that elevated NFR can be seen as a precursor of LFE among workers aged 45 years and older, specifically with regard to early retirement and work disability. Additionally, cross-sectional associations were found between NFR and known determinants of retirement, such as lowered intention and ability to prolong working life, suggesting that NFR plays a role in the LFE decision process. Besides NFR, LFE also seemed to vary based on opportunities to make use of LFE schemes, which might imply that LFE is a result of individual and/or contextual factors. This implication was further strengthened by the finding that factors like gender and unfavourable work conditions seem to affect the strength of the association between NFR and LFE. As such, monitoring NFR among older workers may be used as an early warning system to identify workers with an elevated NFR and offer interventions to reduce NFR and early LFE. Such monitoring should also take into account factors from the demographic, work environment and personal domains. Although such an approach has the potential to facilitate extending labour participation, it should be noted that the effectiveness of measures like worksite social and physical environment interventions or health interventions addressing elevated NFR have yet not been demonstrated (Coffeng et al. 2014; van Berkel et al. 2014).

### Statement

The presented results are based on data of the Maastricht Cohort Study, and are a unique contribution to our understanding on the determinants and consequences of NFR throughout the life span. All earlier studies on this topic which are based on data of the Maastricht Cohort Study have been listed in a supplementary electronic document to this manuscript.

## Electronic supplementary material

Below is the link to the electronic supplementary material.

Supplementary material 1 (DOCX 44 KB)
