# Supplementary material for: Need for recovery and different types of early labour force exit: a prospective cohort study among older workers
Source: Int Arch Occup Environ Health. 2019 Feb 11;92(5):683–97. doi: 10.1007/s00420-019-01404-9 (PMC6556172; doi:10.1007/s00420-019-01404-9)
Supplement: Supplementary file 1 — Supplementary material 1 (DOCX 44 KB) [file 420_2019_1404_MOESM1_ESM.docx]

Publications on the topic of Need for Recovery based on data from the Maastricht Cohort Study

De Raeve L, Vasse RM, Jansen NW, van den Brandt PA, Kant IJ (2007) Mental health effects of changes in psychosocial work characteristics: a prospective cohort study. J Occup Environ 49(8):890-899. https:// doi:10.1097/JOM.0b013e31811eadd3

De Raeve L, Jansen NW, Kant IJ (2007) Health effects of transitions in work schedule, workhours and overtime in a prospective cohort study. Scand J Work Environ Health 33(2):105-113. https:// doi:10.5271/sjweh.1113

De Raeve L, Kant IJ, Jansen NW, Vasse RM, van den Brandt PA (2009) Changes in mental health as a predictor of changes in working time arrangements and occupational mobility: results from a prospective cohort study. J Psychosom Res 66(2):137-145. https://doi: 10.1016/j.jpsychores.2008.05.007.

Fleuren BP, van Amelsvoort LG, de Grip A, Zijlstra FR, Kant IJ (2018) Time takes us all? A two-wave observational study of age and time effects on sustainable employability. Scand J Work Environ Health 44(5):475-484. https://doi: 10.5271/sjweh.3741.

Fleuren BPI, van Amelsvoort LGPM, Zijlstra FRH, de Grip A, Kant IJ (2018) Handling the reflective-formative measurement conundrum: A practical illustration based on sustainable employability. J Clin Epidemiol. Jul 1 pii:S0895-4356(17)31132-0. https://doi: 10.1016/j.jclinepi.2018.07.007.

Gommans F, Jansen N, Stynen D, de Grip A, Kant IJ (2015) The ageing shift worker: a prospective cohort study on need for recovery, disability, and retirement intentions. Scand J Work Environ Health 41(4):356-367. https://doi:10.5271/sjweh.3497.

Gommans FG, Jansen NW, Stynen D, de Grip A, Kant IJ (2015) Need for recovery across work careers: the impact of work, health and personal characteristics. Int Arch Occup Environ Health 88(3):281-295. https://doi: 10.1007/s00420-014-0956-3.

Gommans FG, Jansen NW, Mackey MG, Stynen D, de Grip A, Kant IJ (2016) The Impact of Physical Work Demands on Need for Recovery, Employment Status, Retirement Intentions, and Ability to Extend Working Careers: A Longitudinal Study Among Older Workers. J Occup Environ Med 58(4):e140-151. https://doi: 10.1097/JOM.0000000000000687.

Hoofs H, Jansen NWH, Jansen MWJ, Kant IJ (2017) Monitoring of need for recovery and prolonged fatigue within the working population: Evaluation of reliability and agreement over time. Work 58(3):399-412. https://doi: 10.3233/WOR-172624.

Jansen NW, Kant IJ, van den Brandt PA (2002) Need for recovery in the working population: description and associations with fatigue and psychological distress. Int J Behav Med. 9(4):322-340. https://doi.org/10.1207/S15327558IJBM0904_03

Jansen NW, Kant IJ, Kristensen TS, Nijhuis FJ (2003) Antecedents and consequences of work-family conflict: a prospective cohort study. J Occup Environ Med 45(5):479-491. https:// doi: 10.1097/01.jom.0000063626.37065.e8

Jansen N, Kant IJ, van Amelsvoort L, Nijhuis F, van den Brandt P (2003) Need for recovery from work: evaluating short-term effects of working hours, patterns and schedules. Ergonomics. 10;46(7):664-680. https://doi: 10.1080/0014013031000085662

Jansen NW, Mohren DC, van Amelsvoort LG, Janssen N, Kant IJ (2010) Changes in working time arrangements over time as a consequence of work-family conflict. Chronobiol Int. 27(5):1045-1061. https://doi: 10.3109/07420528.2010.489874.

Kant IJ, Bültmann U, Schröer KA, Beurskens AJ, Van Amelsvoort LG, Swaen GM (2003) An epidemiological approach to study fatigue in the working population: the Maastricht Cohort Study. Occup Environ Med Jun;60 Suppl 1:i32-9. https://doi: 10.1136/oem.60.suppl_1.i32

Kant IJ, Jansen NW, van Amelsvoort LG, Swaen GM, van Leusden R, Berkouwer A (2009) Screening questionnaire Balansmeter proved successful in predicting future long-term sickness absence in office workers. J Clin Epidemiol 62(4):408-414.e2. https://doi: 10.1016/j.jclinepi.2008.07.003.

Lexis MA, Jansen NW, Huibers MJ, van Amelsvoort LG, Berkouwer A, Tjin A Ton G, van den Brandt PA, Kant IJ (2011) Prevention of long-term sickness absence and major depression in high-risk employees: a randomised controlled trial. Occup Environ Med. 68(6):400-7. https://doi: 10.1136/oem.2010.057877.

Lexis MA, Jansen NW, van Amelsvoort LG, Huibers MJ, Berkouwer A, Tjin A Ton G, van den Brandt PA, Kant IJ (2012) Prediction of long-term sickness absence among employees with depressive complaints. J Occup Rehabil 22(2):262-9. https://doi: 10.1007/s10926-011-9334-0.

Mohren DC, Jansen NW, Kant IJ (2010). Need for recovery from work in relation to age: a prospective cohort study. Int Arch Occup Environ Health 83(5):553-61. https://doi: 10.1007/s00420-009-0491-9.

Stynen D, Jansen NW, Kant IJ (2015) The impact of depression and diabetes mellitus on older workers' functioning. J Psychosom Res 79(6):604-13. https://doi:10.1016/j.jpsychores.2015.07.008.

van Amelsvoort LG, Kant IJ, Bültmann U, Swaen GM (2003) Need for recovery after work and the subsequent risk of cardiovascular disease in a working population. Occup Environ Med. Jun;60 Suppl 1:i83-7. http://dx.doi.org/10.1136/oem.60.suppl_1.i83
